# An insecticide-treated bed-net campaign and childhood malaria in Burkina Faso

**DOI:** 10.2471/BLT.14.147702

**Published:** 2015-08-31

**Authors:** Valérie R Louis, Anja Schoeps, Justin Tiendrebéogo, Claudia Beiersmann, Maurice Yé, Marie R Damiba, Guang Y Lu, André H Mbayiha, Manuela De Allegri, Albrecht Jahn, Ali Sié, Heiko Becher, Olaf Müller

**Affiliations:** a Institute of Public Health, Medical School, Heidelberg University, Im Neuenheimer Feld 324, 69120 Heidelberg, Germany.; b Centre de Recherche en Santé de Nouna (CRSN), BP02 Nouna, Burkina Faso.; c Institute of Medical Biometry and Epidemiology, University Medical Center Hamburg–Eppendorf, Hamburg, Germany.

## Abstract

**Objective:**

To investigate if the first national insecticide-treated bed-net campaign in Burkina Faso, done in 2010, was followed by a decrease in childhood malaria in a district with high baseline transmission of the disease.

**Methods:**

We obtained data on the prevalence of *Plasmodium falciparum* parasitaemia in children aged 2 weeks to 36 months from malaria surveys in 2009 and 2011. We assessed morbidity in children younger than 5 years by comparing data from the Nouna health district’s health management information system before and after the campaign in 2010. We analysed mortality data from 2008 to 2012 from Nouna’s health and demographic surveillance system.

**Findings:**

The bed-net campaign was associated with an increase in the reported use of insecticide-treated nets. In 2009, 73% (630/869) of children reportedly slept under nets. In 2011, 92% (449/487) did. The campaign had no effect on the proportion of young children with *P. falciparum* parasitaemia after the rainy season; 52% (442/858) in 2009 and 53% (263/499) in 2011. Cases of malaria increased markedly after the campaign, as did the number of children presenting with other diseases. The campaign was not associated with any changes in child mortality.

**Conclusion:**

The 2010 insecticide-treated net campaign in Burkina Faso was not associated with a decrease in care-seeking for malaria or all-cause mortality in children younger than 5 years. The most likely explanation is the high coverage of nets in the study area before the campaign which could have had an effect on mosquito vectors, limiting the campaign’s impact.

## Résumé

**Objectif:**

Étudier si la première campagne nationale visant à utiliser des moustiquaires imprégnées d'insecticide au Burkina Faso, menée en 2010, a été suivie d'une baisse des cas de paludisme infantile dans un district ayant un taux élevé de transmission de la maladie.

**Méthodes:**

Nous nous sommes procuré des données sur la prévalence de la parasitémie de *Plasmodium falciparum* chez les enfants âgés de 2 semaines à 36 mois à partir des enquêtes sur le paludisme menées en 2009 et 2011. Nous avons évalué la morbidité chez les enfants de moins de 5 ans en comparant les données du système d'information sanitaire du district de Nouna avant et après la campagne de 2010. Nous avons analysé les données sur la mortalité de 2008 à 2012 provenant du système de surveillance sanitaire et démographique de Nouna.

**Résultats:**

La campagne pour l'utilisation de moustiquaires a été associée à une augmentation de l'utilisation de moustiquaires imprégnées d'insecticide. En 2009, d'après l'enquête menée, 73% des enfants (630/869) dormaient sous une moustiquaire. En 2011, ils étaient 92% (449/487). La campagne n'a pas eu d'effet sur la proportion de jeunes enfants présentant une parasitémie de *P. falciparum* après la saison des pluies: ils étaient 52% (442/858) en 2009 et 53% (263/499) en 2011. Les cas de paludisme ont sensiblement augmenté après la campagne, comme le nombre d'enfants atteints d'autres maladies. La campagne n'était associée à aucun changement de la mortalité infantile.

**Conclusion:**

La campagne de 2010 pour l'utilisation de moustiquaires imprégnées d'insecticide au Burkina Faso n'a pas été associée à une diminution des consultations pour paludisme ou de la mortalité, toutes causes confondues, chez les enfants de moins de 5 ans. L'explication la plus probable est liée au taux élevé d'utilisation de moustiquaires dans la zone étudiée avant la campagne, qui pourrait avoir eu un effet sur les moustiques vecteurs de la maladie et avoir limité l'impact de la campagne.

## Resumen

**Objetivo:**

Investigar si la primera campaña nacional de mosquiteros tratados con insecticida en Burkina Faso, que se llevó a cabo en 2010, tuvo como resultado un descenso del paludismo infantil en un distrito con una referencia elevada de transmisión de la enfermedad.

**Métodos:**

Se obtuvieron datos sobre la prevalencia de parasitemia por *Plasmodium falciparum* en niños de edad entre las 2 semanas y los 36 meses de encuestas sobre la malaria de 2009 y 2011. Se evaluó la morbilidad en niños menores de 5 años mediante la comparación de los datos del sistema de gestión de información sanitaria del departamento de salud de Nouna antes y después de la campaña de 2010. Se analizaron los datos de mortalidad desde 2008 hasta 2012 del sistema de vigilancia sanitaria y demográfica de Nouna.

**Resultados:**

La campaña de distribución de mosquiteros se relacionó con un aparente aumento del uso de redes tratadas con insecticida. En 2009, aparentemente el 73% (630/869) de los niños dormía bajo mosquiteros. En 2011, lo hacía el 92% (449/487). La campaña no tuvo efecto en la proporción de niños pequeños con parasitemia por *P. falciparum* después de la temporada de lluvias; el 52% (442/858) en 2009 y el 53% (263/499) en 2011. Los casos de malaria aumentaron notablemente después de la campaña, igual que lo hizo el número de niños que mostraban otras enfermedades. La campaña no se relacionó con ningún cambio en la mortalidad infantil.

**Conclusión:**

La campaña de distribución de mosquiteros tratados con insecticida realizada en 2010 en Burkina Faso no estuvo relacionada con un descenso en la atención médica relacionada con la malaria o la mortalidad a causa de la misma en niños menores de 5 años. La explicación más lógica es la gran cobertura de redes en el área del estudio antes de la campaña, lo que podría haber tenido un efecto en los mosquitos vectores, limitando el impacto de la campaña.

## ملخص

**الغرض:**

استقصاء إذا ما تبع الحملة القومية الأولى لاستخدام الناموسية المعالجة بمبيد حشري في بوركينا فاصو، والتي تم تنفيذها في عام 2010، أي انخفاض في معدل الإصابة بالملاريا عند الأطفال في إحدى المناطق التي تتسم بارتفاع قاعدة انتشار المرض.

**الطريقة:**

حصلنا على بيانات عن انتشار التسمم الدموي بطفيل *المتصورة المنجلية * بين الأطفال الذين تتراوح أعمارهم بين أسبوعين و36 شهراً من خلال المسوح المتعلقة بالملاريا التي تم إجراؤها في عامي 2009 و2011. وأجرينا تقييمًا لمعدل الإصابة بالمرض في الأطفال الذين تقل أعمارهم عن خمس سنوات عن طريق مقارنة البيانات التي تم الحصول عليها من نظام المعلومات المتعلقة بالإدارة الصحية في المنطقة الصحية لمدينة نونا قبل وبعد الحملة في عام 2010. وحللنا البيانات المتعلقة بالوفيات في الفترة من عام 2008 إلى عام 2012 والتي حصلنا عليها من النظام الصحي ونظام الترصد الديموغرافي في مدينة نونا.

**النتائج:**

ارتبطت حملة استخدام الناموسية بزيادة ما أمكن تسجيله من استخدام الناموسيات المعالجة بالمبيد الحشري. ووفقًا لما تم رصده، فقد بلغت نسبة الأطفال الذين ناموا تحت الناموسية 73% (630/869) في عام 2009، بينما بلغت 92% (449/487) في عام 2011. ولم تؤثر الحملة على نسبة الأطفال الصغار المصابين بالتسمم الدموي بطفيل *المتصورة المنجلية * بعد موسم الأمطار، حيث بلغت النسبة 52% (442/858) في عام 2009 و53% (263/499) في عام 2011. وظهر ارتفاعًا ملحوظًا في حالات الإصابة بالملاريا بعد الحملة، مثلما ارتفع عدد الأطفال الذين يعانون من أمراض أخرى. وعليه، فإن الحملة لم ترتبط بأي تغيرات في معدل الوفيات بين الأطفال.

**الاستنتاج:**

لم ترتبط حملة استخدام الناموسية المعالجة بالمبيد الحشري التي تم تنفيذها في عام 2010 في بوركينا فاصو بانخفاض طلب الرعاية الصحية فيما يتعلق بالملاريا أو بمعدل الوفيات لجميع الأسباب في الأطفال الذين تقل أعمارهم عن خمس سنوات. والتفسير الأرجح لذلك هو ارتفاع مستوى التغطية للناموسيات قبل الحملة في المنطقة التي خضعت للدراسة، مما أثَّر على البعوض الناقل للمرض، وأدى إلى الحد من تأثير الحملة. ****

## 摘要

**目的:**

旨在调查布基纳法索在 2010 年展开的第一次全国经杀虫剂处理的蚊帐运动之后，高基线疾病传播地区的儿童疟疾是否减少。

**方法:**

我们从 2009 和 2011 年的疟疾调查中获取了两周到 36 个月大的儿童感染恶性疟原虫寄生虫血症的患病率数据。我们通过比较 2010 年开展该运动前后努纳卫生地区的卫生管理信息系统中的数据，评估了 5 岁以下儿童的死亡率。我们分析了努纳卫生和人口监测系统中 2008 年到 2012 年间的数据。

**结果:**

蚊帐运动与报道的经杀虫剂处理的蚊帐使用量增长相关。据报道，2009 年，73% (630/869) 的儿童睡在蚊帐中。2011 年，92% (449/487) 的儿童睡在蚊帐中。该运动对雨季后感染恶性疟原虫寄生虫血症的儿童比例没有影响；2009 年该比例为 52% (442/858)，2011 年则为 53% (263/499)。该运动开展后，疟疾病例显著增长，同时感染其它疾病的儿童数量也显著增长。该运动与儿童死亡率的变化毫无关联。

**结论:**

布基纳法索在 2010 年开展的经杀虫剂处理的蚊帐运动与 5 岁以下儿童的疟疾求医行为或全因死亡率的降低不相关。最合理的解释是，在该运动开展前，蚊帐在研究区域的高使用率对蚊虫媒介已产生影响，因此使该运动的影响受到限制。

## Резюме

**Цель:**

Выяснить, снизилась ли частота заболевания детей малярией в регионе с высоким базовым уровнем передачи этой инфекции после того, как в 2010 году в Буркина-Фасо была проведена первая национальная кампания по обработке надкроватных сеток инсектицидами.

**Методы:**

Мы получили данные о частоте случаев паразитемии, вызванной *Plasmodium falciparum*, среди детей в возрасте от 2 недель до 36 мес. Данные получены по результатам опросов о заболевании малярией, проведенных в 2009 и 2011 годах. Мы оценили заболеваемость среди детей до 5 лет, сравнивая данные из информационной системы управления отдела здравоохранения региона Ноуна, которые были собраны до проведения кампании в 2010 году и после нее. Были проанализированы сведения о смертности в период с 2008 по 2012 год по данным системы контроля демографической ситуации и системы здравоохранения региона Ноуна.

**Результаты:**

Кампания по обработке сеток сопровождалась ростом числа сообщений об использовании надкроватных сеток, обработанных инсектицидами. По данным отчетов, в 2009 году 73% детей (630 из 869) спали под сетками. В 2011 году их доля составила 92% (449 из 487). Кампания не повлияла на долю малолетних детей, зараженных паразитом *P. falciparum* по окончании сезона дождей: их доля составила 52% в 2009 году (442 из 858) и 53% (263 из 499) в 2011 году. После кампании среди детей наблюдался значительный рост заболеваемости малярией, а также другими заболеваниями. Кампания не ассоциировалась с какими-либо изменениями в уровне детской смертности.

**Вывод:**

Проведенная в Буркина-Фасо в 2010 году кампания по обработке надкроватных сеток инсектицидами не привела к снижению частоты заболевания малярией с обращением родителей за медицинской помощью. Эта кампания также не ассоциировалась со снижением смертности по различным причинам среди детей в возрасте до 5 лет. Наиболее вероятной причиной такого результата может быть тот факт, что в зоне проведения исследования и до начала кампании наблюдался высокий уровень использования надкроватных сеток, что могло повлиять на векторы распространения комаров и ограничить эффект от кампании.

## Introduction

Malaria remains a major threat to the health of some three billion people in 108 countries where the disease is endemic.^1^ According to the World Health Organization (WHO), in 2012 malaria caused 207 million episodes of disease and 627 000 deaths.^2^ About 85% of illness and 90% of deaths attributable to malaria occur in sub-Saharan Africa, with young children being most affected.^3^

After a partially successful malaria eradication campaign in the 1950s and 1960s, the global burden of the disease increased between the 1970s and the 1990s due to a combination of fewer control measures and the development of resistance to antimalarial drugs and insecticides.^3^^,^^4^ After the start of the Roll Back Malaria Initiative in 1998, the global malaria burden decreased again, thanks to the large-scale introduction of interventions such as artemisinin-based combination therapy and insecticide-treated bed nets.^2^^,^^3^ This was associated with a reduction in malaria transmission intensity and the malaria burden in sub-Saharan Africa.^3^^,^^5^ Although surveys indicate that insecticide-treated nets have reduced the malaria burden and all-cause child mortality in Africa,^6^ most recent reports of success in the region have come from islands, the peripheries of endemic areas and smaller countries with substantial external support.^3^^,^^5^^,^^7^ The reported malaria burden has changed little in highly endemic African countries despite increased coverage of insecticide-treated nets.^7^^–^^16^ Moreover, few data are available from countries in sub-Saharan Africa in which the malaria burden is highest.^2^

The aim of our study was to evaluate the effect of the first national campaign designed to achieve universal coverage of insecticide-treated bed nets, which took place in 2010, in a high malaria transmission area of Burkina Faso. In particular, we investigated the effect of the campaign on the prevalence of parasitaemia in young children, childhood morbidity and child mortality.

## Methods

**Fig. 1:**
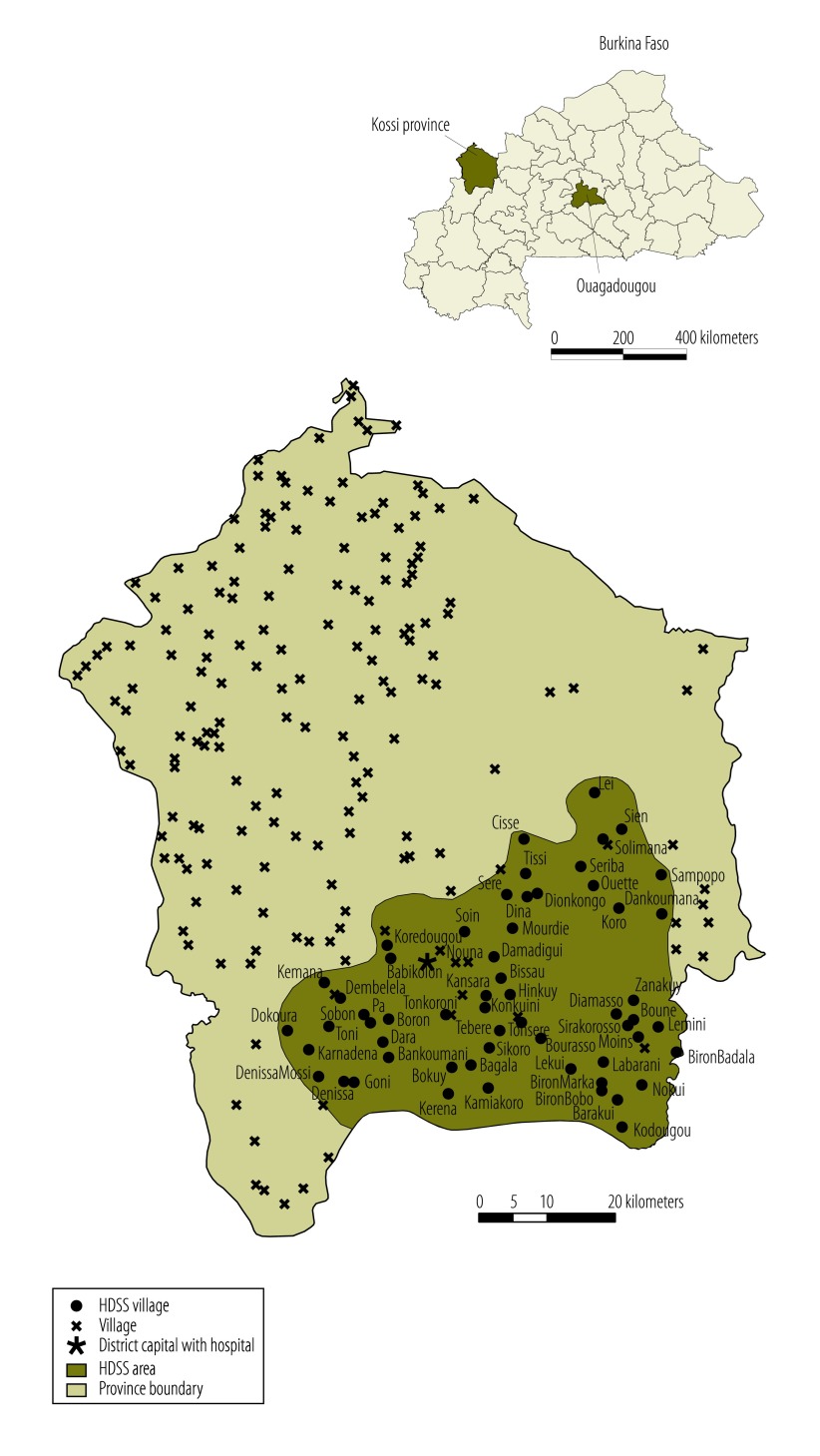
Study area, Nouna health district, Burkina Faso

The study took place in Nouna health district in rural, north-western Burkina Faso, where the annual rainfall varies between 500 and 1000 mm.^17^^,^^18^ The district’s population was 311 000 in 2010 and almost exclusively comprised subsistence farmers. By 2013, it was served by 33 first-line health facilities and one hospital in the district capital Nouna. The Centre de Recherche en Santé de Nouna runs a health and demographic surveillance system (HDSS) site that covers Nouna town and 58 of the 300 villages in Nouna health district (Fig. 1).^19^

HDSS: health and demographic surveillance system.

Notes: Nouna health district covers the same area as Kossi province. The eight villages covered by the four malaria surveys were: Bourasso, Cisse, Kodougou, Koro, Nokui, Seriba, Sikoro and Solimana.

Map produced using shapefiles from ArcGIS (Redlands, CA, United States of America) and location of villages from Institut Géographique du Burkina (Ouagadougou, Burkina Faso).

In the past, malaria was hyperendemic or holoendemic in the area and most cases occurred during and shortly after the rainy season, from July to November.^20^ The disease was also the principle cause of death in young children in the district.^21^^–^^23^ Malaria control interventions were introduced in 2002 and, by 2009, malaria was predominantly mesoendemic in the area.^18^^,^^24^ By early 2010, 59% of households owned at least one insecticide-treated bed net, 67% of pregnant women received intermittent preventive treatment at least once and 34% of children with malaria younger than 5 years received an artemisinin-based combination therapy.^25^ After the national, insecticide-treated net campaign in 2010, 99% of households had at least one net.^26^ Moreover, 66% of young children in the Centre de Recherche en Santé de Nouna study area used nets in the dry season and 98% used them in the rainy season.^27^

### Study data

The effect of the insecticide-treated net campaign on the prevalence of *Plasmodium falciparum* parasitaemia in the blood of young children was assessed from malaria surveys: (i) two cross-sectional surveys of children aged 0.5 to 36 months in eight villages were conducted in June and December 2011, after the campaign; and (ii) two identical surveys were conducted in the two villages in the same months in 2009, before the campaign, as part of a research project comparing child health data from 1999 and 2009.^18^ For both the 2009 and the 2011 survey, 460 children were randomly selected from the Nouna HDSS register for the June survey and followed up in the December survey. The sample size enabled a reduction in malaria parasitaemia prevalence of 15% to be detected with a power of 80% at a significance level of 0.05.

Associations of the campaign with changes in childhood morbidity were assessed by comparing data from Nouna health district’s health management information system for the two years before the campaign (i.e. 2008 and 2009) with data for the two years after the campaign (i.e. 2011 and 2012). For the health management information system, health workers at local health centres sent paper versions of summary statistics on specific diseases to district health teams, who entered data into an electronic database, which were forwarded to regional and national health offices. In this study, we used data from Nouna health district’s database on uncomplicated and severe cases of malaria and, for comparison, on cases of other childhood diseases in children younger than 5 years.

Changes in child mortality were assessed using data for the period 1993 to 2012 from the Nouna HDSS, whose data collection procedures have been described elsewhere.^19^ In brief, fieldworkers visited every household about three times per year and recorded all new births, deaths and migrations and other demographic and health variables.

### Data analysis

The prevalence of *P. falciparum* parasitaemia and the number of reported malaria cases in children before and after the insecticide-treated net campaign were compared descriptively. In addition, the change in the number of recorded malaria cases over the period was compared with the change in reported cases of other diseases in the same age group. Child mortality was estimated in deaths per 1000 person-years for each year between 2008 and 2012. The slight variation in the age distribution between different years caused by a small decrease in the birth rate was corrected for by adjusting the distribution to match that for 2010. To determine whether the campaign affected seasonal mortality, we calculated mortality rates at monthly intervals from January 2008 to December 2012. In addition, mortality rates were also calculated by age group for rural children from the 58 villages covered by the Nouna HDSS and for children from Nouna town.

Ethical approval for the malaria surveys and for the quality assessment of routine data collection at peripheral health facilities in Nouna health district were obtained from the Ethical Committee of Heidelberg University Medical School in Germany and the local ethical committee in Nouna, Burkina Faso. During the surveys, written informed consent was sought from the carers of children involved in the study.

## Results

Data on the prevalence of *P. falciparum* parasitaemia from the four cross-sectional surveys conducted in children aged 0.5 to 36 months are shown in Table 1. Although the reported use of insecticide-treated nets during the night before the survey increased from 73% (630/869) in 2009 to 92% (449/487) in 2011, overall, the prevalence of parasitaemia was similar in the two years, at around 52% (442/858 and 263/499).

**Table 1:** Insecticide-treated bed-net use and *Plasmodium falciparum* parasitaemia in children aged 2 weeks to 36 months, 2009 and 2011, Nouna health district, Burkina Faso

Year	No. (%)					
	Insecticide-treated net use^a^			*P. falciparum* parasitaemia		
	June survey	December survey	Overall	June survey	December survey	Overall
2009	305/460 (66)	325/409 (80)	630/869 (73)	174/453 (38)	268/405 (66)	442/858 (52)
2011	238/254 (94)	211/233 (91)	449/487 (92)	104/262 (40)	159/237 (67)	263/499 (53)

P. falciparum: Plasmodium falciparum.

^a^ Self-reported use of an insecticide-treated net during the night preceding the survey.

Note: The national insecticide-treated bed-net campaign took place in 2010. Surveys were carried out in eight villages in Nouna health district.

**Fig. 2:**
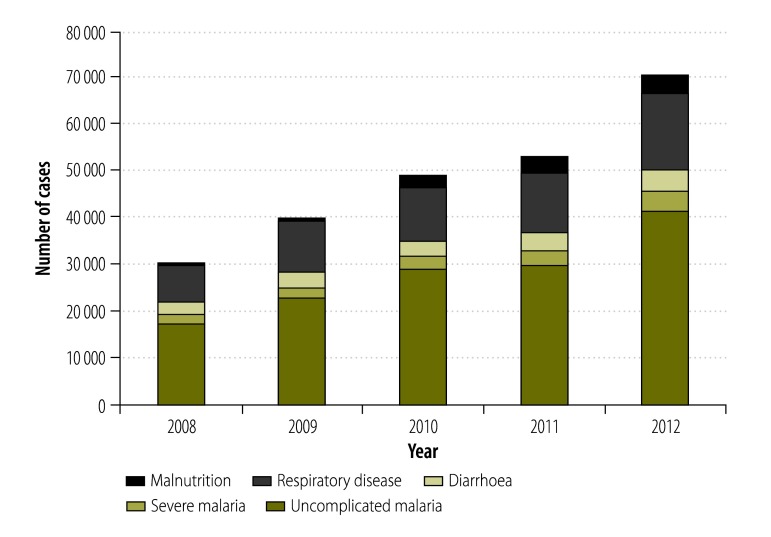
Childhood morbidity by disease, 2008–2012, Nouna health district, Burkina Faso

Notes: The graph shows cases in children younger than 5 years. Asthma and measles cases are not shown due to low numbers.

Data on morbidity in infants and in children younger than 5 years in Nouna health district are shown in Fig. 2 and Table 2. Over the period, 243 057 consultations were reported for all children: 82 116 for infants and 160 941 for children aged 1 to 4 years. Malaria was the leading diagnosis and accounted for more than half of all consultations (154 919); about 10% (13 618) of all malaria cases were categorized as severe. The next leading causes of illness were respiratory disease (59 070), diarrhoea (17 196) and malnutrition (11 684). Both the total number of consultations and the number of consultations for leading causes of illness at health facilities increased continuously from 2008 until 2012. During this period, the number of cases of malaria (both uncomplicated and severe), respiratory disease and diarrhoea roughly doubled in all age groups, whereas the number of malnutrition cases increased by a factor of about 10. The number of uncomplicated malaria cases increased more in older children than infants. The number of measles cases also increased over the study period, but not consistently. There was no clear trend in the low number of asthma cases.

Data source: Health management information system of Nouna health district, Burkina Faso.

**Table 2:** Childhood morbidity, 2008–2012, Nouna health district, Burkina Faso

Disease	Age group, years	No. of cases					
		Year					
		2008	2009	2010	2011	2012	Total
Uncomplicated malaria	< 1	5 676	7 142	8 278	8 818	11 020	40 934
	1 to < 5	11 910	15 874	20 830	21 209	30 544	100 367
Severe malaria	< 1	461	468	593	638	925	3 085
	1 to < 5	1 429	1 522	2 030	2 362	3 190	10 533
Diarrhoea	< 1	916	1 151	1 082	1 358	1 600	6 107
	1 to < 5	1 761	2 059	2 001	2 330	2 938	11 089
Respiratory disease	< 1	3 886	5 491	5 600	6 409	7 304	28 690
	1 to < 5	3 897	5 376	5 825	6 363	8 919	30 380
Malnutrition	< 1	81	158	714	1 122	1 177	3 252
	1 to < 5	302	609	2 131	2 470	2 920	8 432
Measles	< 1	1	0	6	3	17	27
	1 to < 5	0	3	21	4	20	48
Asthma	< 1	11	0	1	8	1	21
	1 to < 5	22	4	18	14	34	92
**Total**	< 1	11 032	14 410	16 274	18 356	22 044	82 116
	1 to < 5	19 321	25 447	32 856	34 752	48 565	160 941

Data sources: Health management information system of Nouna health district, Burkina Faso.

**Fig. 3:**
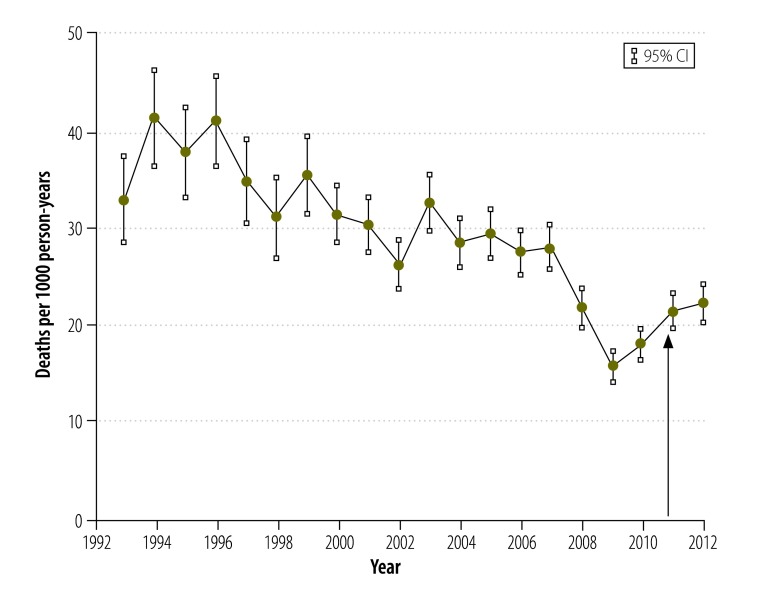
Annual child mortality, Nouna health and demographic surveillance system site, 1993–2012, Burkina Faso

**Fig. 4:**
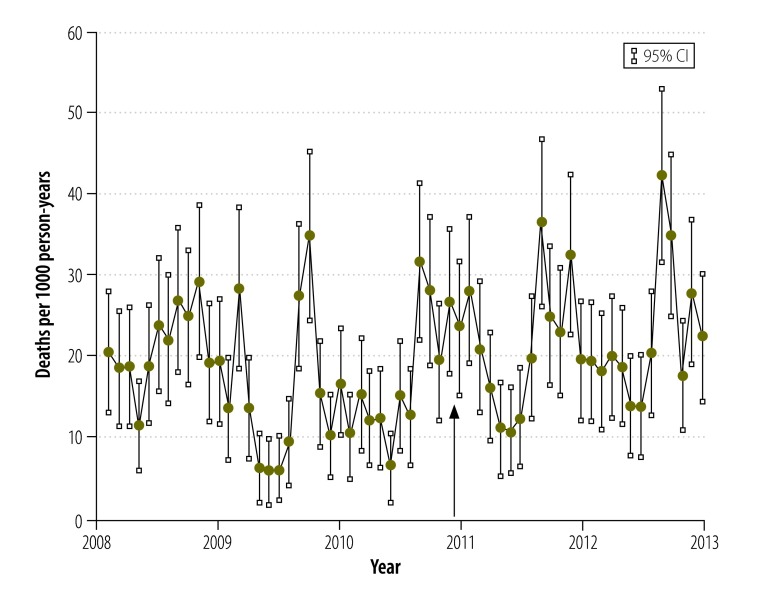
Monthly child mortality, Nouna health and demographic surveillance system site, 2008–2013, Burkina Faso

**Fig. 5:**
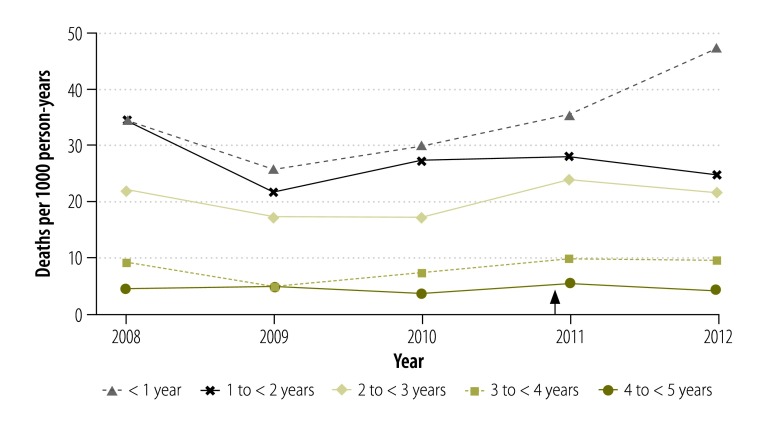
Annual child mortality, by age group, Nouna health and demographic surveillance system site, 2008–2012, Burkina Faso

Note: The arrow indicates the end date of the 1-year-long national insecticide-treated bed-net campaign.

**Fig. 6:**
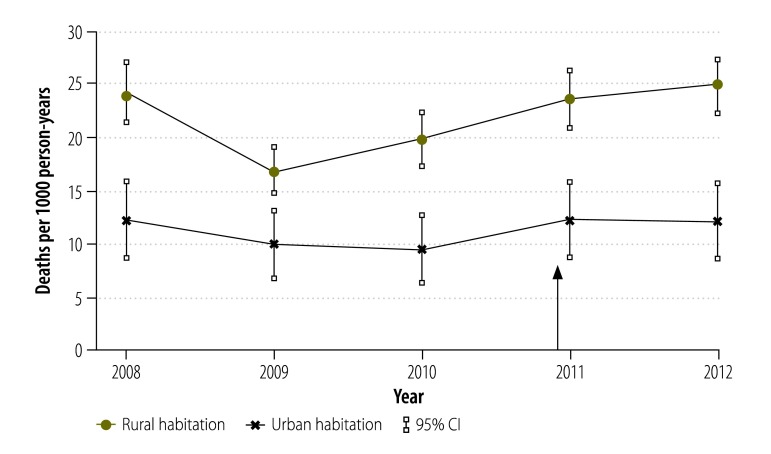
Annual child mortality by habitation, Nouna health and demographic surveillance system site, 2008–2012, Burkina Faso

Overall, there was a substantial decline in all-cause child mortality between 1993 and 2012,^21^^,^^23^ but there was no decrease in the period of interest between 2008 and 2012 (Fig. 3). Moreover, between 2008 and 2012 mortality showed a characteristic seasonal pattern, with no change in the pattern after the insecticide-treated net campaign (Fig. 4). During the period, mortality remained relatively constant in children aged 1 to 4 years but, in infants, there was a slight increasing trend between 2009 and 2012 (Fig. 5). Mortality was significantly lower in Nouna town than the rural neighbouring villages and after 2009 the rural area showed an increasing trend in mortality (Fig. 6).

CI: confidence interval.

Notes: The graph shows deaths in children younger than 5 years. Mortality was age-standardized. The arrow indicates the end date of the 1-year-long national insecticide-treated bed-net campaign.

Data source: Health and demographic surveillance system from Centre de Recherche en Santé de Nouna, Nouna, Burkina Faso.

CI: confidence interval.

Notes: The graph shows deaths in children younger than 5 years. Mortality was age-standardized. The arrow indicates the end date of the 1-year-long national insecticide-treated bed-net campaign.

Data source: Health and demographic surveillance system from Centre de Recherche en Santé de Nouna, Nouna, Burkina Faso.

Data sources: Health and demographic surveillance system from Centre de Recherche en Santé de Nouna, Nouna, Burkina Faso.

CI: confidence interval.

Notes: The graph shows deaths in children younger than 5 years. Mortality was age-standardized. The arrow indicates the end date of the 1-year-long national insecticide-treated bed-net campaign.

Data sources: Health and demographic surveillance system from Centre de Recherche en Santé de Nouna, Nouna, Burkina Faso.

## Discussion

The recent scaling-up of malaria control interventions and increases in international funding have coincided with many reports and publications suggesting that the epidemiology of malaria in sub-Saharan Africa has undergone a major change. However, most of the success stories come from areas with low malaria transmission intensity.^3^^,^^5^^,^^7^^,^^28^^,^^29^ Moreover, the relationship between cause and effect has not always been clear and substantial changes in malaria incidence and prevalence have often preceded expanded coverage by an intervention.^14^^,^^30^^–^^35^

The main finding of our study is that the national insecticide-treated net campaign, which resulted in very high household ownership and use of nets, had no clear effect on the burden of malaria in young children in a highly endemic area. We found that the campaign had not changed the prevalence of malaria parasitaemia; there was even an increase in the annual number of cases. Moreover, there was no substantial change in all-cause child mortality. Recent reports indicate that malaria control programmes in several countries in sub-Saharan Africa with a high malaria burden have also failed to observe any benefits.^7^^–^^16^ In addition, our study found a significant difference in child mortality between urban and rural populations in Burkina Faso, which is in agreement with similar findings in other countries in sub-Saharan Africa.^3^

A few studies have reported on changes in the prevalence of parasitaemia with insecticide-treated net campaigns. One systematic review found that net use was associated with only a small reduction of around 10%.^36^ In highly endemic, mainland Equatorial Guinea, a moderate increase in net use by young children was followed by a slight reduction in the malaria parasite prevalence.^37^ A large controlled study of insecticide-treated net campaigns in Nigeria found lower parasite prevalence in intervention areas, but the results were not conclusive.^38^ In highly endemic areas of western Kenya, the introduction of nets was initially associated with a decrease in parasite prevalence, which increased rapidly thereafter.^39^ In our study area in Nouna, surveys indicated that the prevalence of the malaria parasite in village children declined markedly from 1999 to 2009, when insecticide-treated net coverage was increasing moderately,^18^ whereas the prevalence was unaffected by the 2010 insecticide-treated net campaign. These findings were confirmed by an in-depth longitudinal study in one village.^40^

Most studies on the effect of insecticide-treated net campaigns are based on health management information system data. In Kenya, one study of paediatric inpatients in 17 hospitals showed a mixed pattern of disease incidence over a 10-year period during which coverage with malaria control interventions, including insecticide-treated nets, increased.^8^ Another study done in the same time period showed reductions in malaria admissions to six hospitals but increases in two.^14^ A study from Malawi found some increase in paediatric malaria admissions to four hospitals between 2000 and 2010 despite moderately increased use of insecticide-treated nets.^12^ Similarly, a study at the main hospital in the country was unable to show any change in severe malaria admissions over this period.^10^ In Togo, a mass distribution campaign of insecticide-treated nets was associated with a significant reduction in anaemia in children but also with a 159% increase in malaria incidence in one district.^9^^,^^41^ In addition, a study from Uganda demonstrated that paediatric malaria admissions to five hospitals increased significantly from 1999 to 2009 when there was a massive roll-out of malaria control interventions, including insecticide-treated nets.^15^ Moreover, Ugandan national health statistics showed that the malaria case burden increased from 3.5 million in 2000 to 12.2 million in 2008. However, only 33% of children were sleeping under nets in 2009.^15^ In Zambia, the incidence of malaria increased in one district between 2006 and 2012 despite greater use of insecticide-treated nets.^13^ In contrast, the comprehensive roll-out of malaria control interventions in Rwanda – a hypoendemic country – including an insecticide-treated net campaign, from 2005 was associated with a large reduction in malaria cases.^28^ In our study area, the number of cases of uncomplicated or severe malaria increased after the insecticide-treated net campaign, as did cases of other diseases in young children. The likely explanations are an increase in the population and in parents’ health-care-seeking behaviour, better reporting, service coverage and service quality.

Insecticide-treated nets have been found in a systematic review to reduce all-cause child mortality by about 20% under controlled conditions.^36^ Similar findings were reported in Rwanda and Togo where insecticide-treated net programmes were associated with significant reductions in child mortality.^9^^,^^28^ However, the first studies to demonstrate the efficacy of insecticide-treated nets in sub-Saharan Africa compared coverage of around 80% in intervention groups with 0% in control groups.^36^ Also, in the two studies in Rwanda and Togo, coverage increased from very low to very high levels.^9^^,^^28^ In our study, in contrast, coverage by insecticide-treated nets was already high before the 2010 campaign. Moreover, a decrease in child mortality has been documented in the Nouna study area over the last two decades.^19^^,^^21^^,^^23^

We suggest four potential explanations why the insecticide-treated net campaign in Burkina Faso had no overall beneficial association with malaria burden or mortality in young children. First, the insecticide-treated nets could have been ineffective because of faults in the nets themselves or because the *Anopheles* vector developed resistance to pyrethroid or changed its behaviour. Second, compliance with the insecticide-treated net intervention could have been lower than reported. Third, the efficacy of the insecticide-treated net intervention in an area of high malaria transmission intensity could have been reduced by a saturation effect. Fourth, an unknown biological or social factor could have decreased efficacy.

Currently, there is no evidence that pyrethroid-resistance is an important problem in the study area.^42^ Moreover, studies in sub-Saharan Africa indicate that there is no association between increased resistance of malaria vectors to pyrethroids and decreased efficacy of bed nets.^3^^,^^43^^,^^44^ The 2010 national insecticide-treated net campaign in Burkina Faso seems to have been well implemented and annual surveys in Nouna health district show that net use has remained high.^45^ However, changes in vector behaviour, as reported in other endemic regions, cannot be excluded.^46^

The most likely explanation for the study’s findings is the possibility of a saturation effect with insecticide-treated nets. It has been shown in several sub-Saharan Africa countries that high coverage with nets has a mass effect on the mosquito vector, such that community members who are not sleeping under insecticide-treated nets are also protected from malaria.^3^ At present, the level of coverage needed to provide this mass effect is unknown and it may be that the threshold is far below universal coverage. This hypothesis is supported by mathematical modelling which shows that, when insecticide-treated net use in a community reaches 35% to 65%, individuals not using nets are protected at a similar level as those using them.^47^ More research is needed on the relationship between insecticide-treated net coverage and these saturation effects in areas with different degrees of malaria endemicity.

Our study has strengths and limitations. One strength is that our findings are based on data from a well-established research centre with quality-controlled data collection and management procedures. Moreover, we used data from three different sources which increases the validity of our results.^48^ One limitation is that since the data came partly from the area covered by HDSS and partly from the whole of Nouna health district, the quality of data could have varied. Furthermore, as with any observational study, known or unknown confounders could have influenced the findings, particularly as they were based on health management information system data. For example, increased coverage by a community-based health insurance scheme in Nouna health district could have had an influence. 

However, during the study period, only a small proportion of households participated in the scheme.^49^ Although a few new health facilities have opened in the district in recent years, their presence does not explain the large increase in consultations over a short period of time. Moreover, there was no major variation in the rainfall pattern over the study period (range: 566–763 mm/year). The large increase in the reported number of malnutrition cases may have been due to continuous improvements in primary health-care services in the district in recent years, which have included the expansion of malnutrition treatment services. There were no changes to health management information system procedures during the study period.

In conclusion, the 2010 insecticide-treated net campaign in Burkina Faso was not associated with a decrease in malaria burden or all-cause mortality in children younger than 5 years. This was most likely due to the high baseline coverage of insecticide-treated nets in the study area. Additional tools are needed to reduce the burden of malaria in high-transmission settings.
